# The Society for Implementation Research Collaboration Instrument Review Project: A methodology to promote rigorous evaluation

**DOI:** 10.1186/s13012-014-0193-x

**Published:** 2015-01-08

**Authors:** Cara C Lewis, Cameo F Stanick, Ruben G Martinez, Bryan J Weiner, Mimi Kim, Melanie Barwick, Katherine A Comtois

**Affiliations:** Department of Psychological and Brain Sciences, Indiana University, 1101 E. 10th St., Bloomington, IN 47405 USA; Department of Psychiatry and Behavioral Sciences, Harborview Medical Center, School of Medicine, University of Washington, Box 359911, 325 9th Ave, Seattle, WA 98104 USA; Department of Psychology, University of Montana, 32 Campus Dr., Skaggs Bldg. 362, Missoula, MT 59812 USA; University of North Carolina at Chapel Hill, 1102-C McGavran-Greenberg Hall, 135 Dauer Drive, Campus Box 7411, Chapel Hill, NC 27599-7411 USA; North Carolina Translational and Clinical Sciences Institute, University of North Carolina at Chapel Hill, 160 N. Medical Drive, Brinkhous-Bullitt, 2nd Floor, CB# 7064,, Chapel Hill, NC 27599-7064 USA; Child Health Evaluative Sciences, Research Institute, The Hospital for Sick Children, 555 University Avenue, Toronto, ON M5G 1X8 Canada; Department of Psychology, Virginia Commonwealth University, 806 West Franklin St, Richmond, VA 23220 USA

**Keywords:** Implementation, Dissemination, Instruments, Evidence-based assessment, Psychometrics

## Abstract

**Background:**

Identification of psychometrically strong instruments for the field of implementation science is a high priority underscored in a recent National Institutes of Health working meeting (October 2013). Existing instrument reviews are limited in scope, methods, and findings. The Society for Implementation Research Collaboration Instrument Review Project’s objectives address these limitations by identifying and applying a unique methodology to *conduct* a systematic and comprehensive review of quantitative instruments assessing constructs delineated in two of the field’s most widely used frameworks, *adopt* a systematic search process (using standard search strings), and *engage* an international team of experts to assess the full range of psychometric criteria (reliability, construct and criterion validity). Although this work focuses on implementation of psychosocial interventions in mental health and health-care settings, the methodology and results will likely be useful across a broad spectrum of settings. This effort has culminated in a centralized online open-access repository of instruments depicting graphical head-to-head comparisons of their psychometric properties. This article describes the methodology and preliminary outcomes.

**Methods:**

The seven stages of the review, synthesis, and evaluation methodology include (1) setting the scope for the review, (2) identifying frameworks to organize and complete the review, (3) generating a search protocol for the literature review of constructs, (4) literature review of specific instruments, (5) development of an evidence-based assessment rating criteria, (6) data extraction and rating instrument quality by a task force of implementation experts to inform knowledge synthesis, and (7) the creation of a website repository.

**Results:**

To date, this multi-faceted and collaborative search and synthesis methodology has identified over 420 instruments related to 34 constructs (total 48 including subconstructs) that are relevant to implementation science. Despite numerous constructs having greater than 20 available instruments, which implies saturation, preliminary results suggest that few instruments stem from gold standard development procedures. We anticipate identifying few high-quality, psychometrically sound instruments once our evidence-based assessment rating criteria have been applied.

**Conclusions:**

The results of this methodology may enhance the rigor of implementation science evaluations by systematically facilitating access to psychometrically validated instruments and identifying where further instrument development is needed.

**Electronic supplementary material:**

The online version of this article (doi:10.1186/s13012-014-0193-x) contains supplementary material, which is available to authorized users.

## Background

Identification of psychometrically strong instruments for the field of implementation science is a high priority in the United States, as underscored in a recent National Institute of Health working meeting (October 2013; Rabin et al., unpublished).^a^ Reliable and valid instruments are critical to scientific advancement as they allow for careful collection, expression, and comparison of results of observation and experimentation [1]. Unfortunately, poor-quality instruments have slowed the discovery and application of evidence-based implementation strategies for supporting widespread delivery of evidence-based care. Many new fields face instrumentation challenges until consensus builds around high-quality measures of key constructs. Without consensus, informative, applicable instrumentation will remain slow and hindered by duplicative efforts and incommensurable results. For an in-depth discussion of instrumentation issues in implementation science, see Martinez et al. [2].

Existing instrument review efforts within the field of Dissemination and Implementation Science (DIS) focus on individual constructs such as readiness for change (e.g., [3]) constructs that predict specific implementation outcomes such as adoption [4] and on broader reviews of multi-level domains [5]. Other instrument review efforts such as the Grid-Enabled Measures Project (GEM; [6]; https://www.gem-beta.org/public/wsoverview.aspx?cat=8&wid=11&aid=0) engage researchers and stakeholders in populating and evaluating an online repository of measures. Thus far, review efforts reveal that few instruments have undergone systematic development and are psychometrically strong. These instrument review efforts represent important contributions as they inform the state of measurement quality in the field and support a significant need for additional research in this area.

Despite these instrument review efforts, three important gaps remain. First, no existing instrument reviews include a comprehensive array of constructs relevant for DIS. A comprehensive review of constructs is important to guide instrument selection and development and then to facilitate identification of constructs that are implicated in successful implementation. Second, existing methodologies for instrument reviews are narrowly focused and only provide limited psychometric assessments of the instruments. Specifically, Chaudoir et al.’s instrument review focused only on predictive validity [5]. Although predictive validity is critical to the identification of key constructs, in the absence of also establishing reliability and/or content and construct validity, predictive validity is only marginally informative. Further, Chor et al.’s work provided dichotomous (yes/no) conclusions about the psychometric validation of instruments without providing an indication of the process for this determination [4]. These limitations of existing instrument review methodologies must be addressed to support quality measurement in this field. Third, no protocol exists to systematically develop a compendium or repository of instruments for widespread use. An open-source resource would facilitate simultaneous access to instruments and comparison between instruments with respect to their psychometric strength. A centralized online database that is searchable and provides head-to-head comparisons of instrument psychometric properties would be a significant step forward for the field.

### The current project: aims and objectives

The Society for Implementation Research Collaboration (SIRC; formerly known as the Seattle Implementation Research Collaborative)^b^ Instrument Review Project (IRP) has established a methodology for instrument review to address these gaps by a) conducting a systematic and comprehensive review of quantitative instruments assessing constructs delineated in two of the field’s most highly cited frameworks, the Consolidated Framework for Implementation Research (CFIR; [7]) and the Implementation Outcomes Framework (IOF; [8]); b) adopting and applying a systematic search process (using standard search strings); c) engaging an international team of experts to assess the full range of psychometric criteria (reliability, construct validity, and criterion validity); and d) building a centralized online, open access, evolving repository of instruments depicting graphical head-to-head comparisons of their psychometric properties. Existing instrument review and repository efforts are summarized and compared in a separate manuscript that highlights their unique contributions and the gaps in the field that the SIRC IRP seeks to fill (see Rabin et al., unpublished). In this article, we describe the SIRC IRP methodology and summarize preliminary results of the 420+ instruments that have been identified according to the following:

- the number of instruments identified for each of the 48 DIS constructs (including the 13 subconstructs; CFIR and IOF),
- the rigor underlying instrument development,
- whether the construct was explicitly defined in the original article,
- the year and field in which the instrument was created,
- the stakeholder targeted by the instrument,
- settings in which the instrument has been used, and
- the number of published studies reporting use of the instrument (bibliometric data).

The findings from this methodology will inform a pressing research agenda by identifying priorities for measurement development. Moreover, the online repository will position those invested in advancing the field of implementation science (e.g., researchers and stakeholders: agency leaders, purveyors, decision makers in service provider organizations) to engage in rigorous evaluation of their implementation initiatives by providing online access to instruments, associated peer reviewed articles, and information regarding their psychometric properties. Although the resulting repository is geared towards implementation of psychosocial interventions in mental health and health-care settings to be consistent with the focus of SIRC, the repository is designed to promote the use of instruments across disciplines that will be useful to researchers and stakeholders implementing evidence-based practices across a broad spectrum of settings.

## Methods

### Step 1: defining the scope of the project

The instrument review protocol and development of the repository focuses on quantitative instruments used in the implementation of evidence-based practices or innovations in mental health, health care, and school settings. To adhere to this scope, we developed the following two criteria for identifying relevant instruments: a) if the instrument assesses some aspect of implementation science with regard to settings where mental health interventions are used, it will be regarded as relevant; and b) if an instrument can be easily adapted to make its subject pertinent to the mental health field, it will be deemed relevant (e.g., only the name of the intervention, population, or setting would need to be changed within the instrument).

### Step 2: selecting theoretical frameworks to guide the review

Our team prioritized identifying a theoretical framework that could guide identification and organization of the instruments according to key DIS constructs. Although there are over 60 guiding frameworks for DIS ([9]; e.g., PARiHS [10], DoI [11], PRISM [12]), there is little agreement and little empirical evidence on which constructs are more important for planning and evaluation [13]. Few theoretical frameworks come close to comprehensively outlining the diverse array of constructs and domains implicated. However, two of the most highly cited frameworks were selected to categorize and organize instruments: (1) the CFIR [3] and (2) the IOF [4].

The CFIR was an obvious first choice as it fits with our goal to be as comprehensive as possible. Specifically, the CFIR is a meta-theoretical framework generated to address the lack of uniformity in the DIS theory landscape that minimizes overlap and redundancies in available frameworks, separates ideas that had been formerly seen as inextricable, and creates a uniform language for the domains and constructs of DIS. Our team conceptualizes the CFIR constructs as potential predictors, moderators, and mediators or “drivers” of DIS outcomes. Despite the fairly comprehensive nature of the CFIR, it is limited in that clearly defined outcomes for DIS are missing. DIS outcomes are distinct from clinical treatment and service system outcomes. Implementation outcomes are typically measured in implementation activities, can advance understanding of the implementation processes, enhance efficiency in implementation research, and pave the way for studies of the comparative effectiveness of implementation strategies [8]. To address this limitation, our team identified a second framework put forth by Proctor et al.’s work delineating “implementation outcomes” [8]. The isolation and concrete operationalization of implementation outcomes, separate from service and client outcomes, was a unique and important addition to the literature (Table 1). This added focus may be critical in future research seeking to understand the temporal relations between constructs. Our team conceptualizes implementation outcomes, such as *penetration* and *sustainability*, as dependent variables in a DIS process and, therefore, as integral constructs warranting inclusion in a comprehensive review of DIS instruments. A detailed review of the theories and frameworks summarized here can be found elsewhere [9].

**Table 1:** **Listing of included and excluded constructs from the organizing frameworks**

	**Construct**	**Included**	**Excluded**
CFIR domains			
Characteristics of individuals	Knowledge and beliefs about the intervention	X	
	Individual stage of change	X	
	Individual identification with organization	X	
	Other personal attributes	X	
	Self-efficacy	X	
Inner setting	Culture	X	
	Implementation climate (IC)	X	
	IC: tension for change^a^	X	
	IC: compatibility^a^	X	
	IC: relative priority^a^	X	
	IC: organizational incentives and rewards^a^	X	
	IC: goals and feedback^a^	X	
	IC: learning climate^a^	X	
	Networks and communications	X	
	Readiness for implementation (RI)	X	
	RI: leadership engagement^a^	X	
	RI: available resources^a^	X	
	RI: access to knowledge and information^a^	X	
	Structural characteristics	X	
Intervention characteristics	Adaptability	X	
	Complexity	X	
	Cost	X	
	Design quality and packaging	X	
	Evidence strength and quality	X	
	Intervention source	X	
	Relative advantage	X	
	Trialability	X	
Outer setting	Cosmopolitanism	X	
	External policy and incentives	X	
	Patient needs and resources	X	
	Peer pressure	X	
Process	Engaging	X	
	Engaging: opinion leaders^a^	X	
	Engaging: formally appointed internal^a^	X	
	Implementation leaders^a^		
	Engaging: champions^a^	X	
	Engaging: external change agents^a^	X	
	Executing	X	
	Planning	X	
	Reflecting and evaluating	X	
Implementation outcomes framework			
Service outcomes	Effectiveness		X
	Efficiency		X
	Equity		X
	Patient-centeredness		X
	Safety		X
	Timeliness		X
Client outcomes	Function		X
	Satisfaction	X	
	Symptomology		X
Implementation outcomes	Acceptability	X	
	Adoption	X	
	Appropriateness	X	
	Cost	X	
	Feasibility	X	
	Fidelity	X	
	Penetration	X	
	Sustainability	X	
Total		48	8

There are 34 main constructs with a total of 48 including subconstructs. Adapted from Damschroder et al. [7] (http://www.implementationscience.com/content/4/1/50/additional/) and Proctor et al. [8].

*CFIR* Consolidated Framework for Implementation Research.

^a^Subconstructs.

In sum, by combining the two frameworks, the resulting repository would include instruments based on a comprehensive listing of constructs implicated at the inception of an implementation project, throughout the early stages of an implementation, as well as those thought to contribute to the success of an implementation initiative. Constructs are defined here as factors inside domains that predict, moderate, or mediate DIS as well as implementation outcomes. The following domains guide review of the DIS instrument literature: *characteristics of the intervention, outer setting, inner setting, characteristics of the individuals involved in implementation, process, implementation outcomes, and client outcomes* (see Table 1).

### Step 3: generating a search protocol for the literature review of constructs

Utilizing the CFIR and IOF, a scoping review of the DIS literature was conducted, broadly, in search of instruments and related articles that purportedly measured each of the 48 constructs (including subconstructs). Scoping reviews are a useful first step to inform the parameters of subsequent systematic reviews [14]. In our scoping review process, we completed searches of PsycINFO and Web of Science to explore the landscape of DIS instruments and identify those relevant to mental health. This first pass of the literature on DIS constructs resulted in identification of 105 instruments.

This exploratory stage was integral to setting search parameters to guide the subsequent review. This task was undertaken using the help of a trained information specialist. From this scoping review, a publication date parameter was set to include only those articles published after 1985 to maximize the relevance of instruments identified given how recently the science of dissemination and implementation has emerged. Drawing upon the work of Straus et al. [15], McKibbon et al. [16], and Powell et al. [17] who published helpful search strings for DIS literature reviews, a core set of search word strings that reflected the parameters of the project were identified (see core search strings in Additional file 1). Titles and abstracts were examined to exclude obviously irrelevant articles. Articles that survived the title and abstract review were then reviewed more thoroughly with special attention paid to the articles’ method sections. In addition, the articles’ references were reviewed and articles that appeared likely to yield new instruments were accessed.

Once an instrument was identified as relevant, it was sent to the project leads (i.e., C.C.L., C.S., R.G.M., and B.J.W.) for verification. Disagreements were resolved through careful review and consensus among our core workgroup. Disagreements were most often a result of issues of homonymy and synonymy as described in Martinez et al. [2], failure of the author to define the construct of interest, and misalignment (or multiple alignment) of the targeted construct with the constructs delineated by the organizing frameworks. In each case, at least two core workgroup members reviewed all available material and took one of the following actions: place the instrument within its most relevant construct, place the instrument within multiple constructs for ease of access, or exclude the instrument altogether.

The initial construct reviews were replicated by a team of research assistants (RA) at a second site. Each instrument author was contacted to obtain the full-length instrument in the event it was not included in the original article and to request permission to post the instrument under the password protection of the SIRC website for members to access. This process sought to improve the yield of available instruments to populate the developing repository.

Concurrent with the review of published literature, a snowball sampling email procedure was used to locate instruments in preparation or otherwise unpublished instruments. This was particularly important for preventing the creation of redundant instruments and extends this methodology beyond that of a typical systematic review. The snowball sampling technique accessed DIS stakeholders through relevant email LISTSERV (e.g., SIRC membership; Association of Behavioral and Cognitive Therapies Dissemination and Implementation Science Special Interest Group) and personal contacts. DIS-related websites across disciplines with a particular focus on mental health and health care were also reviewed for instruments or related papers and authors were subsequently contacted. Stakeholders who received emails from our group were encouraged to share the email request for DIS instruments with colleagues in the field.

### Step 4: the literature review of specific instruments—extending beyond a systematic review

In the instrument review phase, we systematically compiled all information regarding each identified instrument, particularly with respect to the development of psychometrics and any data relevant to the evidence-based assessment (EBA) criteria described below in step 5. This step is a significant deviation from a typical systematic review protocol, but a necessary and effective innovation for our methodology to evaluate and synthesize the literature and produce a decision aid for researchers and stakeholders. As with the construct reviews, PsycINFO and Web of Science served as the primary databases for the instrument review. The instrument name written in quotations (e.g., “Treatment Acceptability Rating Form”) served as the primary search string; the search was then limited by drawing upon the core set of search terms outlined in Additional file 1. Specific instrument reviews were replicated by a second RA. When completed, all documents pertaining to a single instrument were compiled and combined into a single PDF (henceforth referred to as a *packet*) in preparation for the quality assessment phase in step 6: data extraction and rating.

### Step 5: development of the evidence-based assessment rating criteria

In order to ensure that all identified instruments are evaluated for their psychometric qualities using a relevant system that is amenable to a large-scale collaborative effort, we developed an evidence-based assessment rating criteria. These criteria were derived from the EBA criteria of Hunsley and Mash’s earlier work that focused on standardized patient outcome measures [18] and from the work of Terwee et al. [19]. These criteria will ensure that all identified instruments are evaluated for their psychometric qualities using a standardized system. To reduce rater subjectivity and enhance inter-rater reliability, the criterion anchors needed to be especially concrete. The main modifications included increasing the number of anchors (from 3–5) to promote variability of the ratings.

To maximize the utility and relevance of the EBA criteria for the purposes of DIS, the first draft was sent to 106 expert DIS scientists, members of the SIRC Network of Expertise. We obtained 60 responses containing rich conceptual (e.g., how to include DIS-specific criterion) and practical (e.g., how to improve the likelihood that anchors would be selected reliably) feedback. All 60 responses were reviewed and integrated by the project’s core workgroup. The second draft of the EBA rating criteria was then sent to local experts in classical test theory and test development. A third version of the EBA criteria emerged from further revising the anchors in accordance with the expert feedback. In total, this final version of the EBA rating system included six criteria reflecting: norms, reliability information, criterion (predictive) and construct (structural) validity information, responsiveness (sensitivity to change), and usability (assessed by length). Each criterion included a five-point anchoring system for rating ranging from “0” or “no evidence” to “4” or “excellent evidence” (see Table 2 for the final version of the EBA).

**Table 2:** **Evidence-based assessment criteria**

**Criterion**	**Description**
Reliability information	
0	None (N): *α* values are not yet available or are only available for subscales
1	Minimal/emerging (M): *α* values of <0.60
2	Adequate (A): *α* values of 0.60–0.69
3	Good (G): *α* values of 0.70–0.79
4	Excellent (E): *α* values of ≥0.80
NA	Internal consistency measures are not applicable for this measure or classical test theory anchors are not appropriate, results reported using item response theory
Structural validity	
0	None (N): no exploratory or confirmatory analysis has yet been performed nor any Item Response Theory tests of (uni-)dimensionality have been conducted, or percent variance explained is not reported
1	Minimal/emerging (M): the sample consisted of less than five times the number of items and an exploratory factor analysis explained less than 25% of the variance
2	Adequate (A): the sample consisted of less than five times the number of items but is less than 100 in total and an exploratory factor analysis explained less than 50% of the variance or a confirmatory factor analysis revealed an RMSEA of 0.08 to 0.05 or CFI = 0.90 to 0.95
3	Good (G): the sample consisted of five times the number of items and is greater than or equal to 100 in total or the sample consisted of five to seven times the number of items but is less than 100 in total and in either case an exploratory factor analysis explained less than 50% of the variance or a confirmatory factor analysis revealed an RMSEA of 0.05 to 0.03 or CFI = 0.95 to 0.97
4	Excellent (E): the sample consisted of seven times the number of items and is greater than 100 in total and an exploratory analysis explained greater than 50% of the variance or a confirmatory factor analysis revealed an RMSEA of <0.03 or CFI > 0.97
Criterion (predictive) validity information	
0	None (N): predictive validity not yet tested or failed to be detected in evaluation
1	Minimal/emerging (M): evidence of small correlation (α range: 0.10 to 0.29) between measure and scores on another test (measuring a distinct construct of interest or outcome) administered at some point in the future
2	Adequate (A): evidence of medium correlation (*α* range: 0.30 to 0.49) between measure and scores on another test (measuring a distinct construct of interest or outcome) administered at some point in the future
3	Good (G): evidence of strong correlation (*α* range: 0.50 to 1.00) between measure and scores on another test (measuring a distinct construct of interest or outcome) administered at some point in the future
4	Excellent (E): evidence of medium-strong correlation (*α* range: 0.30 or higher) between measure and scores on at least two other tests (measuring a distinct construct of interest or outcome) administered at some point in the future
Norms	
0	None (N) none: norms are not yet available
1	Minimal/emerging (M): measures of central tendency and distribution for the total score (and subscales if relevant) based only on a small (*n* < 30) sample are available
2	Adequate (A): measures of central tendency and distribution for the total score (and subscales if relevant) based on a moderate (*n* = 30–49) sample are available
3	Good (G): measures of central tendency and distribution for the total score (and subscales if relevant) based on a medium (*n* = 50–99) sample are available
4	Excellent (E): measures of central tendency and distribution for the total score (and subscales if relevant) based on a large (*n* > 100) sample are available
Responsiveness (sensitivity to change)	
0	None (N): the measure has either not been administered both pre- and post-implementation to evaluate sensitivity to change or it has been administered and it did not demonstrate responsiveness (change) across an implementation process
1	Minimal/emerging (M): the measure demonstrated change over time based on a small (*n* < 50) sample
2	Adequate (A): the measure demonstrated either clinically or statistically significant change over time based on a medium sample (*n* > 50 but <100)
3	Good (G): the measure demonstrated change over time reflective of both clinically and statistically significant change based on a large sample (*n* > 100)
4	Excellent (E): the measure demonstrated both clinically and statistically significant change over time based on at least two large (*n* > 100) samples
Usability (measure length)	
0	None (N): the measure is not in the public domain
1	Minimal (M): the measure has greater than 100 items
2	Adequate (A): the measure has greater than 50 items but fewer than 100
3	Good (G): the measure has greater than 10 items but fewer than 50
4	Excellent (E): the measure has fewer than 10 items

### Step 6: data extraction and rating instruments

The data extraction phase is ongoing to capture the most up-to-date public information on the instruments included in the repository. In this phase, the data is extracted by independent reviewers (RAs) using a standardized, piloted extraction procedure. Specifically, data referencing EBA-relevant information is highlighted and labeled by an RA for each article in every packet (which contains the instrument, the source article, and all associated peer reviewed publications in which the instrument is used). The purpose is to have well-trained RAs systematically complete the data extraction to promote ease of rating by the volunteer task force member (i.e., expert implementation scientist). Each packet is randomly assigned to an in-house advanced RA (often a PhD-level research scientist) plus one task force member to be rated for its psychometric strength and usability using the EBA criteria. Modeled after the work of Terwee et al. [19], we employed a “worst score counts” methodology. This is an intentionally conservative approach that also facilitates reliability in the rating process. Cohen’s kappa is computed to assess inter-rater reliability, and rating discrepancies are resolved through consensus among the core workgroup.

**Figure 1:**
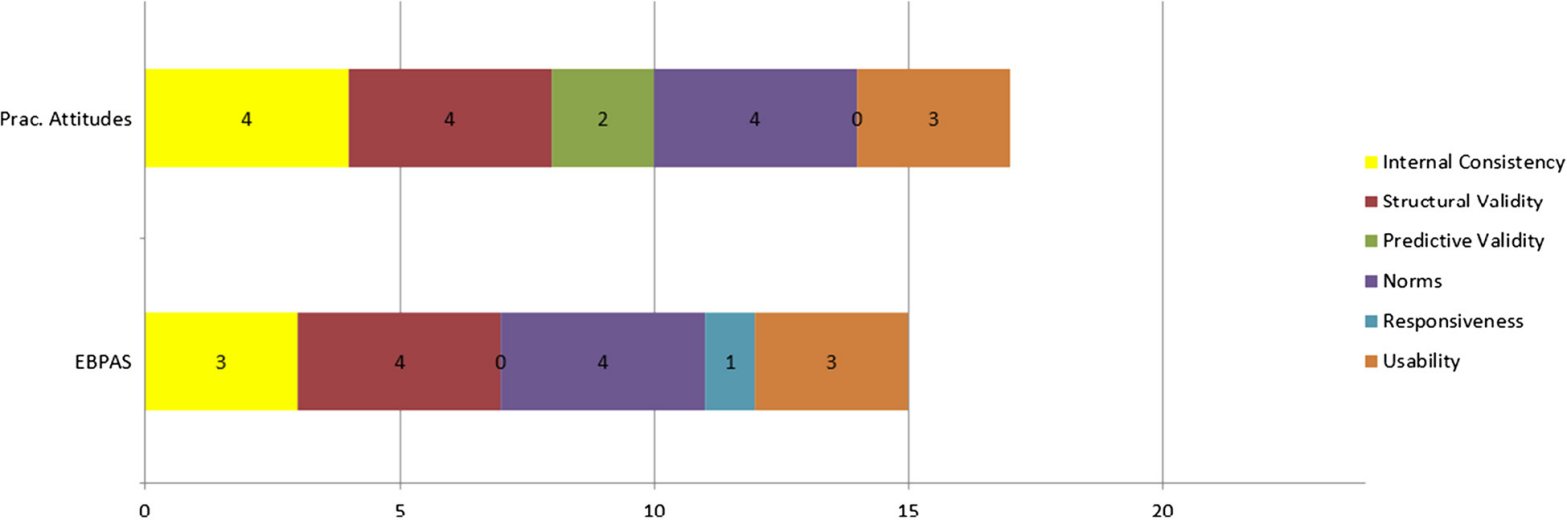
**A head-to-head comparison of the evidence-based practice attitudes scale (EBPAS) and the practitioner’s attitudes towards treatment manuals scale psychometric properties.** Total possible score equals 24. Criteria rated 0 to 4: 0 = “none”, 1 = “minimal”, 2 = “adequate”, 3 = “good”, and 4 = “excellent” [20,21].

Figure 1 presents an illustration of the EBA criteria application and the resulting graphical displays of criterion scores. In this figure, two measures of evidence-based practice acceptability were evaluated according to the EBA rating process. As depicted in Figure 1, the Evidence-based Practice Attitudes Scale (EBPAS), a 15-item self-report measure that assesses “mental health provider attitudes toward adoption of evidence-based practice” [20], is directly compared with Addis and Krasnow’s 17-item self-report measure that assesses practitioners’ attitudes towards treatment manuals [21]. Using the worst score counts methodology and available data, the ratings reveal that the EBPAS is of high psychometric quality overall. Both instruments appear to have garnered strong psychometric properties including established structural validity (i.e., EFA/PCA analyses have accounted for more than 50% of variance), available norms, and fewer than 50 items. However, readers have the capacity to determine for themselves which qualities are most important (e.g., responsiveness versus predictive validity). The EBPAS has demonstrated stronger internal consistency and is more responsive (i.e., sensitive) to change. Conversely, Addis and Krasnow’s [21] measure appears to have more consistently predicted criterion measures. Important to note is that the EBPAS has demonstrated predictive validity in previous studies (e.g. [22]) but not in all. This is a prime example of how the worst score count methodology operates and affects the interpretation of instrument comparisons.

### Step 7: population of the website repository

Once both sets of ratings are attained, data are converted into a head-to-head graphical comparison that depicts the relative and absolute psychometric strength of an instrument relative to others for that construct (see Figure 1). This information is contained in the website repository alongside the instrument and links to all relevant literature. This step is integral for researchers and other stakeholders to efficiently judge the state of instrumentation for each construct.

### Preliminary results and discussion

#### Preliminary results

Despite identifying over 420 instruments across the 48 DIS constructs (including subconstructs), we uncovered critical gaps in DIS instrumentation. Preliminary results highlight constructs for which few to no instruments exist (see Table 3). Specifically, our review methodology revealed no instruments for the following constructs, many of which fall within CFIR’s outer setting domain: *complexity of the intervention, intervention design quality and packaging, intervention source, external policies and incentives, peer pressure, tension for change, goals and feedback, formally appointed internal implementation leaders, and engaging champions.* Many other constructs appear to have only one or two instruments available (e.g., *compatibility*, *relative priority*). These preliminary results suggest that there is a great need for instrument development to advance DIS, particularly in the critical domain of outer setting. In the absence of outer setting measures, the field will be challenged to identify the role that these constructs play in successful implementation across different contexts. Interestingly, despite the recently renewed NIH program announcement explicitly highlighting their interest in instrument-related proposals, they have received few proposals centered on instrument development (David Chambers DPhil, personal communication, October 24, 2013).

**Table 3:** **Summary of preliminary results**

**Domain**	**Construct**	**Instruments per construct**	**Stage of dev.**	**Stage of dev.**	**Percentage of instruments with definition**	**Number of articles in packet**
		***N***	***M***	**Mode**	***N*** **(%)**	***M*** **(SD)**
Implementation outcomes	Acceptability	46	3.11	4	33 (71.74%)	4.41 (4.11)
	Adoption	24	3.58	1	21 (87.50%)	1.58 (1.52)
	Appropriateness	7	1.00	1	3 (42.86%)	1.29 (1.10)
	Feasibility	14	1.00	1	6 (42.86%)	1.57 (1.46)
	Penetration	5	2.40	1	5 (100%)	2.60 (2.08)
	Sustainability	9	2.44	1	6 (66.67%)	1.67 (1.48)
Total		105			74	
Average		17.5	2.26	1	70.48%	2.19 (1.96)
Intervention characteristics	Adaptability	1	4.00	1, 2, 3, 4	1 (100%)	1.00 (1.00)
	Complexity	4	3.50	1	3 (75.00%)	1.00 (0.75)
	Design quality and packaging	0	0.00	0	0 (0.00%)	0.00 (0.00)
	Evidence strength and quality	4	1.75	1	3 (75.00%)	1.00 (0.75)
	Intervention source	0	0.00	0	0 (0.00%)	0.00 (0.00)
	Relative advantage	7	2.43	1	5 (71.43%)	1.00 (0.71)
	Trialability	3	4.00	1, 6, 7, 8	2 (66.67%)	1.00 (0.67)
Total		19			14	
Average		2.71	2.24	1	73.68%	0.71 (0.55)
Outer setting	Cosmopolitanism	1	3.00	2, 6, 8	0 (0.00%)	4.00 (0.00)
	External policy and incentives	0	0.00	0	0 (0.00%)	0.00 (0.00)
	Patient needs and resources	3	4.67	6	2 (66.67%)	1.00 (0.67)
	Peer pressure	0	0.00	0	0 (0.00%)	0.00 (0.00)
Total		4			2	
Average		1	1.92	6	50.00%	1.25 (0.17)
Inner setting	Combined	9	4.44	1	8 (88.89%)	9.22 (8.20)
	Culture	10	5.00	1	10 (100%)	4.44 (3.95)
	Implementation climate (IC)	15	5.60	1	14 (93.33%)	6.93 (6.47)
	IC: tension for change	0	0.00	0	0 (0.00%)	0.00 (0.00)
	IC: compatibility	1	8.00	1, 2, 3, 4, 5, 6, 7, 8	1 (100%)	11.00 (0.00)
	IC: relative priority	1	2.00	1 and 6	1 (100%)	3.00 (0.00)
	IC: organizational incentives and rewards	4	5.75	1,6,7,8	4 (100%)	3.00 (2.25)
	IC: goals and feedback	0	0.00	0	0 (0.00%)	0.00 (0.00)
	IC: learning climate	14	4.64	1	14 (100%)	9.29 (8.62)
	Networks and communications	11	4.36	1	9 (81.82%)	5.17 (4.40)
	Readiness for implementation (RI)	16	3.38	1	13 (81.25%)	2.74 (2.59)
	RI: leadership engagement	4	5.75	1, 6, 7, 8	4 (100%)	4.50 (3.38)
	RI: available resources	2	3.00	1, 6	2 (100%)	2.50 (1.25)
	RI: access to knowledge and information	1	8.00	1, 2, 3, 4, 5, 6, 7, 8	1 (100%)	7.00 (0.00)
	Structural characteristics	2	3.50	1, 6	2 (100%)	7.50 (3.75)
Total		90			83	
Average		6.00	4.23	1	92.22%	5.09 (2.99)
Characteristics of individuals	Knowledge and beliefs about the intervention	52	3.84	2	31 (5.36%)	4.48 (4.40)
	Individual stage of change	6	2.83	1	5 (83.33%)	3.00 (2.50)
	Individual identification with the organization	4	3.50	1, 4	3 (16.67%)	2.83 (2.36)
	Other personal attributes	34	2.65	1	27 (5.26%)	3.89 (3.79)
	Self-Efficacy	4	3.75	1	4 (100%)	2.75 (2.06)
Total		98			70	
Average		19.6	3.31	2	71.43%	3.39 (3.02)
Process	Engaging	0	0.00	0	0 (0.00%)	1.00 (0.00)
	Engaging: opinion leaders	0	0.00	0	0 (0.00%)	5.67 (3.78)
	Engaging: formally appointed internal implementation leaders	0	0.00	0	0 (0.00%)	0.00 (0.00)
	Engaging: champions	0	0.00	0	0 (0.00%)	0.00 (0.00)
	Engaging: external change agents	1	7.00	1, 2, 3, 5, 6, 7, 8	1 (100%)	10.00 (0.00)
	Executing	1	5.00	1, 2, 6, 7, 8	1 (100%)	2.00 (0.00)
	Planning	21	2.14	1	13 (61.90%)	7.25 (6.99)
	Reflecting and evaluating	20	0.03	0	9 (45.00%)	2.70 (2.22)
Total		54			24	
Average		6.75	1.77	1	44.44%	3.58 (1.62)
Client outcomes	Satisfaction	10	2.80	1, 3, 4, 5	4 (40.00%)	4.43 (4.11)

Stages of dev. means the stages of development through which the instrument passed based on an eight-stage coding system describe in the text. It is important to note that these stages are not necessarily linear, meaning that an instrument need not pass through stage one to enter stage two and so forth. Rather, instruments received a point for any of the stages the instrument passed through. Finally, these ratings are reflective of the instruments’ quality at its inception (i.e., based on its source article) and are not necessarily indicative of the instruments’ current psychometric strength.

Numerous constructs have 20 or more available instruments (e.g., *acceptability*, *adoption*, *organizational context*, *culture*, *implementation climate*, *knowledge and beliefs about the intervention*, *other personal attributes*, *planning*, *reflecting*, and *evaluating*), suggesting saturation. However, without readily available information on what exists nor the psychometric properties and associated decision making tools, DIS researchers and stakeholders may continue to develop instruments in these seemingly saturated areas or select poorly constructed instruments that will hinder scientific progress. It is important for researchers and stakeholders to carefully consider the applicability of available instruments to promote cross-study comparisons, which is a necessary process for building the DIS knowledge base.

**Figure 2:**
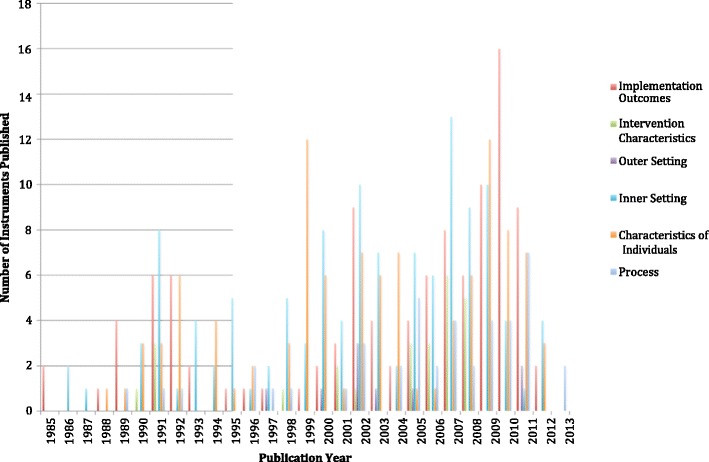
**Timeline of instrument development.**

Figure 2 depicts the timeline across which identified instruments were developed (“year developed” is based on the year in which the original article was published). That is, based on our search parameter (i.e., beginning in 1985), less than one quarter (23.17%) of all identified instruments were developed prior to 1999 (14-year period), whereas one quarter (25.61%) of instruments have been developed since 2009 (4-year period), reflecting the growth of DIS in recent years. Notably, and perhaps not surprisingly, over one third (34.90%) of instruments for implementation outcomes have been developed since the seminal paper by Proctor et al. was published [8]. Proctor et al. articulated a research agenda for DIS outcome evaluations that appears to have positively influenced instrument development.

Table 4 summarizes the six discrete fields from which the instruments emerged. The majority of instruments tapping *implementation outcomes* emerged from subfields of Psychology. Instruments tapping *intervention characteristics* stem from Psychology and Public Health or Government research. *Inner setting* instruments emerged from the previously mentioned fields, although more significantly from Organizational, Workplace, and Business literatures. Instruments tapping *characteristics of individuals*, *process*, and *client outcomes* were generated from a range of fields including those listed previously but also Medicine and Education. The breakdown of fields from which the identified instruments were generated suggests that Psychology and its subfields have contributed immensely to the evaluation of DIS, representing a higher average number of instruments than any other field across constructs (*M* = 3.91). Notably, the discipline from which the instruments emerged was consistent with the strengths of each field.

**Table 4:** **Fields from which instruments originated**

**Domain**	**Construct**	**Education**	**Psychology**	**IT**	**Public health**	**Medicine**	**Organizational**
Implementation outcomes	Acceptability	8 (18.61%)	29 (67.44%)	1 (2.33%)	3 (6.98%)	2 (4.65%)	0 (0.00%)
	Adoption	5 (33.33%)	5 (33.33%)	1 (6.67%)	3 (20.00%)	1 (6.67%)	0 (0.00%)
	Appropriateness	0 (0.00%)	3 (60.00%)	1 (20.00%)	1 (20.00%)	0 (0.00%)	0 (0.00%)
	Feasibility	1 (9.09%)	7 (63.64%)	0 (0.00%)	3 (27.27%)	0 (0.00%)	0 (0.00%)
	Penetration	0 (0.00%)	2 (40.00%)	0 (0.00%)	3 (60.00%)	0 (0.00%)	0 (0.00%)
	Sustainability	0 (0.00%)	1(20.00%)	0 (0.00%)	4 (80.00%)	0 (0.00%)	0 (0.00%)
Intervention characteristics	Adaptability	0 (0.00%)	1 (100.00%)	0 (0.00%)	0 (0.00%)	0 (0.00%)	0 (0.00%)
	Complexity	0 (0.00%)	1 (25.00%)	1 (25.00%)	2 (50.00%)	0 (0.00%)	0 (0.00%)
	Design quality and packaging	0 (0.00%)	0 (0.00%)	0 (0.00%)	0 (0.00%)	0 (0.00%)	0 (0.00%)
	Evidence strength and quality	0 (0.00%)	3 (75.00%)	0 (0.00%)	1 (25.00%)	0 (0.00%)	0 (0.00%)
	Intervention source	0 (0.00%)	0 (0.00%)	0 (0.00%)	0 (0.00%)	0 (0.00%)	0 (0.00%)
	Relative advantage	0 (0.00%)	2 (40.00%)	1 (20.00%)	2 (40.00%)	0 (0.00%)	0 (0.00%)
	Trialability	0 (0.00%)	0 (0.00%)	1 (33.33%)	2 (66.67%)	0 (0.00%)	0 (0.00%)
Outer setting	Cosmopolitanism	0 (0.00%)	0 (0.00%)	0 (0.00%)	1 (100.00%)	0 (0.00%)	0 (0.00%)
	External policy and incentives	0 (0.00%)	0 (0.00%)	0 (0.00%)	0 (0.00%)	0 (0.00%)	0 (0.00%)
	Patient needs and resources	0 (0.00%)	2 (66.67%)	0 (0.00%)	1 (33.33%)	0 (0.00%)	0 (0.00%)
	Peer pressure	0 (0.00%)	0 (0.00%)	0 (0.00%)	0 (0.00%)	0 (0.00%)	0 (0.00%)
Inner setting	Combined	0 (0.00%)	1 (20.00%)	0 (0.00%)	4 (80.00%)	0 (0.00%)	0 (0.00%)
	Culture	0 (0.00%)	1 (50.00%)	0 (0.00%)	1 (50.00%)	0 (0.00%)	0 (0.00%)
	Implementation climate	0 (0.00%)	3 (23.08%)	1 (7.69%)	2 (15.39%)	1 (7.69%)	6 (46.15%)
	IC: tension for change	0 (0.00%)	0 (0.00%)	0 (0.00%)	0 (0.00%)	0 (0.00%)	0 (0.00%)
	IC: compatibility	0 (0.00%)	0 (0.00%)	1 (100.00%)	0 (0.00%)	0 (0.00%)	0 (0.00%)
	IC: relative priority	0 (0.00%)	0 (0.00%)	0 (0.00%)	1 (100.00%)	0 (0.00%)	0 (0.00%)
	IC: organizational incentives and rewards	0 (0.00%)	1 (33.33%)	1 (33.33%)	0 (0.00%)	0 (0.00%)	1 (33.33%)
	IC: goals and feedback	0 (0.00%)	0 (0.00%)	0 (0.00%)	0 (0.00%)	0 (0.00%)	0 (0.00%)
	IC: learning climate	0 (0.00%)	3 (21.43%)	0 (0.00%)	0 (0.00%)	0 (0.00%)	11 (78.57%)
	Networks and communications	0 (0.00%)	0 (0.00%)	0 (0.00%)	0 (0.00%)	0 (0.00%)	10 (100.00%)
	Readiness for implementation	1 (8.33%)	1 (8.33%)	0 (0.00%)	1 (8.33%)	5 (41.67%)	4 (34.33%)
	RI: leadership engagement	0 (0.00%)	2 (100.00%)	0 (0.00%)	0 (0.00%)	0 (0.00%)	0 (0.00%)
	RI: available resources	0 (0.00%)	0 (0.00%)	0 (0.00%)	0 (0.00%)	0 (0.00%)	1 (100.00%)
	RI: access to knowledge and information	0 (0.00%)	0 (0.00%)	1 (100.00%)	0 (0.00%)	0 (0.00%)	0 (0.00%)
	Structural characteristics	0 (0.00%)	1 (100.00%)	0 (0.00%)	0 (0.00%)	0 (0.00%)	0 (0.00%)
Characteristics of individuals	Knowledge and beliefs about the intervention	1 (2.00%)	38 (76.00%)	1 (2.00%)	0 (0.00%)	6 (12.00%)	4 (8.00%)
	Individual stage of change	0 (0.00%)	4 (80.00%)	0 (0.00%)	0 (0.00%)	0 (0.00%)	1 (20.00%)
	Individual identification with the organization	2 (40.00%)	0 (0.00%)	0 (0.00%)	1 (20.00%)	1 (20.00%)	1 (20.00%)
	Other personal attributes	0 (0.00%)	11 (42.31%)	0 (0.00%)	0 (0.00%)	9 (34.62%)	6 (23.08%)
	Self-efficacy	0 (0.00%)	4 (100.00%)	0 (0.00%)	0 (0.00%)	0 (0.00%)	0 (0.00%)
Process	Engaging	0 (0.00%)	0 (0.00%)	0 (0.00%)	0 (0.00%)	0 (0.00%)	0 (0.00%)
	Engaging: opinion leaders	0 (0.00%)	0 (0.00%)	0 (0.00%)	0 (0.00%)	0 (0.00%)	0 (0.00%)
	Engaging: formally appointed internal implementation leaders	0 (0.00%)	0 (0.00%)	0 (0.00%)	0 (0.00%)	0 (0.00%)	0 (0.00%)
	Engaging: champions	0 (0.00%)	0 (0.00%)	0 (0.00%)	0 (0.00%)	0 (0.00%)	0 (0.00%)
	Engaging: external change agents	0 (0.00%)	0 (0.00%)	0 (0.00%)	0 (0.00%)	1 (100.00%)	0 (0.00%)
	Executing	0 (0.00%)	1 (100.00%)	0 (0.00%)	0 (0.00%)	0 (0.00%)	0 (0.00%)
	Planning	0 (0.00%)	5 (25.00%)	0 (0.00%)	5 (25.00%)	2 (10.00%)	8 (40.00%)
	Reflecting and evaluating	2 (14.29%)	11 (78.57%)	0 (0.00%)	0 (0.00%)	1 (7.14%)	0 (0.00%)
Client outcomes	Satisfaction	1 (10.00%)	2 (20.00%)	0 (0.00%)	0 (0.00%)	4 (40.00%)	3 (30.00%)
*M* # of instruments across constructs		0.46	3.91	0.24	0.89	0.72	1.22

Fifty-eight instruments did not have an identifiable field of origin. “Psychology” includes clinical, counseling, community, school, sports, social, developmental, and forensic. “Medicine” includes psychiatry, VA, nursing, and pediatrics. “Organizational” includes workplace and business. Public health also includes government agency.

Tables 5 and 6 reflect the stakeholders targeted by each instrument and the contexts in which the instruments have been used, respectively. Across domains, the majority of instruments were developed to target the service provider rather than the service director, supervisor, or consumer. However, measures of *intervention characteristics* and *process* targeted stakeholders in the “other” category, encompassing a range of general staff as well as researchers. In line with the field from which the instruments originated and the scope of the review, the majority of instruments have since been used in mental health settings.

**Table 5:** **Stakeholders targeted by instruments**

**Domain**	**Construct**	**Director** ***N*** **(%)**	**Supervisor** ***N*** **(%)**	**Provider** ***N*** **(%)**	**Consumer** ***N*** **(%)**	**Other** ***N*** **(%)**
Implementation outcomes	Acceptability	0 (0.00%)	1 (1.96%)	31 (60.78%)	16 (31.37%)	3 (5.88%)
	Adoption	4 (16.67%)	5 (20.83%)	11 (45.83%)	2 (8.33%)	8 (33.33%)
	Appropriateness	0 (0.00%)	1 (14.29%)	3 (42.86%)	1 (14.29%)	2 (28.57%)
	Feasibility	1 (7.14%)	1 (7.14%)	7 (50.00%)	6 (42.86%)	1 (7.14%)
	Penetration	0 (0.00%)	2 (40.00%)	1 (20.00%)	1 (20.00%)	1 (20.00%)
	Sustainability	0 (0.00%)	0 (0.00%)	4 (44.44%)	2 (22.22%)	3 (33.33%)
Intervention characteristics	Adaptability	0 (0.00%)	0 (0.00%)	1 (100%)	1 (100%)	0 (0.00%)
	Complexity	0 (0.00%)	0 (0.00%)	3 (75.00%)	0 (0.00%)	1 (25.00%)
	Design quality and packaging	0 (0.00%)	0 (0.00%)	0 (0.00%)	0 (0.00%)	0 (0.00%)
	Evidence strength and quality	0 (0.00%)	1 (20.00%)	3 (60.00%)	1 (20.00%)	0 (0.00%)
	Intervention source	0 (0.00%)	0 (0.00%)	0 (0.00%)	0 (0.00%)	0 (0.00%)
	Relative advantage	0 (0.00%)	0 (0.00%)	4 (57.14%)	1 (14.29%)	3 (42.86%)
	Trialability	0 (0.00%)	0 (0.00%)	2 (66.67%)	0 (0.00%)	1 (33.33%)
Outer setting	Cosmopolitanism	0 (0.00%)	0 (0.00%)	0 (0.00%)	0 (0.00%)	1 (100%)
	External policy and incentives	0 (0.00%)	0 (0.00%)	0 (0.00%)	0 (0.00%)	0 (0.00%)
	Patient needs and resources	1 (33.33%)	0 (0.00%)	2 (66.67%)	0 (0.00%)	0 (0.00%)
	Peer pressure	0 (0.00%)	0 (0.00%)	0 (0.00%)	0 (0.00%)	0 (0.00%)
Inner setting	Combined	1 (11.11%)	2 (22.22%)	5 (55.55%)	1 (11.11%)	1 (11.11%)
	Culture	2 (25.00%)	3 (37.50%)	5 (62.50%)	2 (25.00%)	0 (0.00%)
	Implementation climate	1 (6.67%)	10 (66.67%)	5 (33.33%)	4 (26.67%)	1 (6.67%)
	IC: tension for change	0 (0.00%)	0 (0.00%)	0 (0.00%)	0 (0.00%)	0 (0.00%)
	IC: Compatibility	0 (0.00%)	0 (0.00%)	0 (0.00%)	0 (0.00%)	1 (100%)
	IC: Relative priority	1 (100%)	1 (100%)	1 (100%)	0 (0.00%)	0 (0.00%)
	IC: Organizational incentives and rewards	0 (0.00%)	2 (50.00%)	1 (25.00%)	0 (0.00%)	1 (25.00%)
	IC: goals and feedback	0 (0.00%)	0 (0.00%)	0 (0.00%)	0 (0.00%)	0 (0.00%)
	IC: learning climate	0 (0.00%)	6 (42.86%)	1 (7.14%)	7 (50.00%)	0 (0.00%)
	Networks and communications	0(0.00%)	3 (25.00%)	4 (33.33%)	6 (50.00%)	0 (0.00%)
	Readiness for implementation	2 (10.53%)	0 (0.00%)	3 (15.79%)	5 (26.32%)	10 (52.63%)
	RI: leadership engagement	1(25.00%)	4 (100%)	2 (50.00%)	1 (25.00%)	0 (0.00%)
	RI: available resources	0 (0.00%)	2 (100%)	1 (50.00%)	0 (0.00%)	0 (0.00%)
	RI: access to knowledge and information	0 (0.00%)	0 (0.00%)	0 (0.00%)	0 (0.00%)	1 (100%)
	Structural characteristics	0 (0.00%)	0 (0.00%)	1 (50.00%)	0 (0.00%)	1 (50.00%)
Characteristics of individuals	Knowledge and beliefs about the intervention	1 (1.79%)	2 (3.57%)	40 (71.43%)	8 (14.29%)	7 (12.50%)
	Individual stage of change	0 (0.00%)	0 (0.00%)	4 (66.67%)	1 (16.67%)	1 (16.67%)
	Individual identification with the organization	(0.00%)	0 (0.00%)	0 (0.00%)	5 (83.33%)	1 (16.67%)
	Other personal attributes	0 (0.00%)	0 (0.00%)	8 (21.05%)	18 (47.37%)	12 (31.58%)
	Self-efficacy	0 (0.00%)	0 (0.00%)	2 (50.00%)	1 (25.00%)	1 (25.00%)
Process	Engaging	0 (0.00%)	0 (0.00%)	1 (100%)	0 (0.00%)	0 (0.00%)
	Engaging: opinion leaders	0 (0.00%)	0 (0.00%)	0 (0.00%)	0 (0.00%)	3 (100%)
	Engaging: formally appointed internal implementation leaders	0 (0.00%)	0 (0.00%)	0 (0.00%)	0 (0.00%)	0 (0.00%)
	Engaging: champions	0 (0.00%)	0 (0.00%)	0 (0.00%)	0 (0.00%)	0 (0.00%)
	Engaging: external change agents	0 (0.00%)	1 (100%)	0 (0.00%)	0 (0.00%)	0 (0.00%)
	Executing	0 (0.00%)	0 (0.00%)	1 (100%)	0 (0.00%)	0 (0.00%)
	Planning	0 (0.00%)	5 (17.86%)	5 (17.86%)	5 (17.86%)	17 (60.71%)
	Reflecting and evaluating	0 (0.00%)	2 (10.00%)	9 (45.00%)	1 (5.00%)	10 (50.00%)
Client outcomes	Satisfaction	0 (0.00%)	0 (0.00%)	4 (28.57%)	5 (35.71%)	6 (42.86%)

“Other” represents general staff or researchers.

**Table 6:** **Contexts in which instruments have been used**

**Domain**	**Construct**	**Health care** ***N*** **(%)**	**Workplace** ***N*** **(%)**	**Mental illness/substance abuse** ***N*** **(%)**	**Education** ***N*** **(%)**	**Other** ***N*** **(%)**
Implementation outcomes	Acceptability	3 (5.88%)	0 (0.00%)	28 (54.90%)	22 (43.14%)	0 (0.00%)
	Adoption	2 (8.33%)	6 (25.00%)	5 (20.83%)	5 (20.83%)	6 (25.00%)
	Appropriateness	1 (14.29%)	1 (14.29%)	2 (28.57%)	1 (14.29%)	2 (28.57%)
	Feasibility	5 (35.71%)	0 (0.00%)	8 (57.14%)	3 (21.43%)	3 (21.43%)
	Penetration	1 (20.00%)	1 (20.00%)	3 (60.00%)	2 (40.00%)	0 (0.00%)
	Sustainability	1 (11.11%)	3 (33.33%)	4 (44.44%)	0 (0.00%)	1 (11.11%)
Intervention characteristics	Adaptability	1 (100%)	0 (0.00%)	0 (0.00%)	1 (100%)	0 (0.00%)
	Complexity	1 (25.00%)	0 (0.00%)	1 (25.00%)	0 (0.00%)	2 (50.00%)
	Design quality and packaging	0 (0.00%)	0 (0.00%)	0 (0.00%)	0 (0.00%)	0 (0.00%)
	Evidence strength and quality	0 (0.00%)	0 (0.00%)	4 (100%)	0 (0.00%)	0 (0.00%)
	Intervention source	0 (0.00%)	0 (0.00%)	0 (0.00%)	0 (0.00%)	0 (0.00%)
	Relative advantage	1 (14.29%)	0 (0.00%)	2 (28.57%)	0 (0.00%)	4 (57.14%)
	Trialability	1 (33.33%)	0 (0.00%)	0(0.00%)	0 (0.00%)	2 (66.67%)
Outer setting	Cosmopolitanism	0 (0.00%)	1 (100%)	0 (0.00%)	0 (0.00%)	0 (0.00%)
	External policy and incentives	0 (0.00%)	0 (0.00%)	0 (0.00%)	0 (0.00%)	0 (0.00%)
	Patient needs and resources	1 (33.33%)	0 (0.00%)	2 (66.67%)	0 (0.00%)	0 (0.00%)
	Peer pressure	0 (0.00%)	0 (0.00%)	0 (0.00%)	0 (0.00%)	0 (0.00%)
Inner setting	Combined	6 (66.67%)	1 (11.11%)	1 (11.11%)	0 (0.00%)	1 (11.11%)
	Culture	1 (12.50%)	5 (62.50%)	1 (12.50%)	0 (0.00%)	1 (12.50%)
	Implementation climate	4 (26.67%)	7 (46.67%)	2 (13.33%)	1 (6.67%)	3 (20.00%)
	IC: tension for change	0 (0.00%)	0 (0.00%)	0 (0.00%)	0 (0.00%)	0 (0.00%)
	IC: compatibility	0 (0.00%)	0 (0.00%)	0 (0.00%)	0 (0.00%)	1 (100%)
	IC: relative priority	0 (0.00%)	0 (0.00%)	0 (0.00%)	1 (100%)	0 (0.00%)
	IC: organizational incentives and rewards	0 (0.00%)	2 (50.00%)	1 (25.00%)	0 (0.00%)	1 (25.00%)
	IC: goals and feedback	0 (0.00%)	0 (0.00%)	0 (0.00%)	0 (0.00%)	0 (0.00%)
	C: learning climate	0 (0.00%)	13 (92.86%)	0 (0.00%)	1 (7.14%)	0 (0.00%)
	Networks and communications	1 (6.25%)	10 (62.50%)	4 (25.00%)	0 (0.00%)	1 (6.25%)
	Readiness for implementation	5 (26.32%)	1 (5.26%)	3 (15.79%)	1 (5.26%)	9 (47.37%)
	RI: leadership engagement	0 (0.00%)	1 (25.00%)	3 (75.00%)	0 (0.00%)	0 (0.00%)
	RI: available resources	0 (0.00%)	1 (50.00%)	1 (50.00%)	0 (0.00%)	0 (0.00%)
	RI: access to knowledge and information	0 (0.00%)	0 (0.00%)	0 (0.00%)	0 (0.00%)	1 (100%)
	Structural characteristics	0 (0.00%)	0 (0.00%)	1 (50.00%)	0 (0.00%)	1 (50.00%)
Characteristics of individuals	Knowledge and beliefs about the intervention	3 (5.36%)	4 (7.14%)	37 (66.07%)	6 (10.71%)	8 (14.29%)
	Individual stage of change	1 (16.67%)	1 (16.67%)	3 (50.00%)	0 (0.00%)	1 (16.67%)
	Individual identification with the organization	0 (0.00%)	5 (83.33%)	0 (0.00%)	0 (0.00%)	1 (16.67%)
	Other personal attributes	2 (5.26%)	17 (44.74%)	7 (18.42%)	0 (0.00%)	12 (31.58%)
	Self-efficacy	1 (20.00%)	0 (0.00%)	2 (40.00%)	1 (20.00%)	1 (20.00%)
Process	Engaging	0 (0.00%)	0 (0.00%)	1 (100%)	0 (0.00%)	0 (0.00%)
	Engaging: opinion leaders	0 (0.00%)	0 (0.00%)	0 (0.00%)	0 (0.00%)	3 (100%)
	Engaging: formally appointed internal implementation leaders	0 (0.00%)	0 (0.00%)	0 (0.00%)	0 (0.00%)	0 (0.00%)
	Engaging: champions	0 (0.00%)	0 (0.00%)	0 (0.00%)	0 (0.00%)	0 (0.00%)
	Engaging: external change agents	1 (100%)	0 (0.00%)	0 (0.00%)	0 (0.00%)	0 (0.00%)
	Executing	0 (0.00%)	0 (0.00%)	1 (100%)	0 (0.00%)	0 (0.00%)
	Planning	2 (7.14%)	5 (17.86%)	5 (17.86%)	0 (0.00%)	17 (60.71%)
	Reflecting and evaluating	1 (5.00%)	1 (5.00%)	8 (40.00%)	1 (5.00%)	9 (45.00%)
Client outcomes	Satisfaction	2 (14.29%)	1 (7.14%)	4 (28.57%)	1 (7.14%)	6 (42.86%)

Bibliometric data available for each of the identified instruments (see Table 3) makes it possible to deduce which instruments have been perceived favorably by researchers conducting DIS via publication counts for each instrument. This information is of course confounded by the year in which the instrument was developed and thus should be interpreted with caution. To date, instruments tapping *inner setting* are the most frequently used and published. Notably, *compatibility* instruments have an average of 11 publications, followed by *combined* instruments (e.g., culture and climate, average of 9.22 instruments). *External change agent* instruments have an average of 10 published articles. *Implementation outcomes* are receiving greater attention in the literature; despite having far fewer publications, there is steady growth over the recent years.

With data extraction and psychometric ratings ongoing (step 6), we can nevertheless provide a preliminary account of the quality of the identified instruments. Across the 48 constructs (including subconstructs), an average of 71% included explicit construct definitions. This suggests that the construct validity of approximately one quarter of the instruments, which is based on careful operationalization of constructs according to their theoretical underpinnings, is questionable. In the absence of explicit construct definitions, use of identified instruments by other teams requires investigators to make assumptions about the instrument’s construct validity based on available items, which may be challenging given the potential overlapping nature of constructs within domains (e.g., the construct of *appropriateness* is often used synonymously with *perceived fit*, *relevance*, *compatibility*, *suitability*, *usefulness*, and *practicability*; [8]). Until consensus among constructs and terms is achieved [23], this practice may compromise the generalizability of study findings.

A second set of preliminary results suggests that in general, the identified DIS instruments are of poor quality. Specifically, we developed a coding system to rate the stages of systematic development through which each instrument should progress. Eight stages were identified based on seminal work of Walsh and Betz [24]: (1) construct is defined, (2) initial items are generated by a group of experts, (3) pilot test of items with representative sample, (4) validity and reliability tests conducted based on pilot testing, (5) instrument is refined based on pilot results, (6) refined instrument is administered to the targeted sample, (7) validity and reliability tests are performed, and (8) psychometric properties are reported. Each instrument was coded such that 1 point was assigned for each aforementioned stage through which the instrument progressed as reported in the original articles. Table 3 indicates that on average, the instruments identified did not even pass through three (of a possible eight) full stages of “proper” instrument development based on our coding system. These preliminary results suggest that the systematic development and psychometric characteristics of the body of instruments available in DIS is weak at best. However, these findings need to be substantiated by our rigorous psychometric evaluation, which is currently underway, in order to place confidence in these observations.

### A comparison of SIRC’s methodology to existing reviews and repositories

To date, using this multi-faceted and collaborative search, synthesis, and evaluation methodology, SIRC’s IRP has identified over 420 instruments tapping 48 constructs (including subconstructs) relevant to DIS. Use of this methodology, which combines systematic review techniques with email snowball sampling (to identify instruments in progress) and ongoing review of the latest publications, has resulted in a more comprehensive DIS instrument database than previous efforts. Specifically, although Chaudoir et al. [5] employed a systematic review of key DIS domains (i.e., structural, organizational, provider, patient, and innovation, as opposed to constructs: e.g., intervention adaptability, external policy, and incentives), they identified only 62 instruments which is substantially fewer than the 420+ instruments revealed by the SIRC methodology. We posit that the low number of instruments identified by Chaudoir et al. is due to the exclusion of instruments that assess implementation outcomes, arguably the most critical domain of DIS constructs to date, and due to the fewer number of domains included in their review.

Moreover, our review methodology is unique because unlike previous reviews, all literature pertaining to each instrument has been identified to enable accurate conclusions about individual instrument quality. Previous efforts to employ a collaborative instrument review process, notably the GEM [6], do not systematically locate all available literature to rate the quality of the instruments. Rather, the GEM approach encourages website users to provide their own ratings regardless of user knowledge of the extant literature.

### Implications

This multi-faceted methodology has potential long-term implications for DIS. Upon creation of the repository, researchers and stakeholders will have a relevant and useful resource for identifying available and psychometrically sound DIS instruments, thereby reducing the need to create “homegrown” instruments (i.e., relevant for one-time use; [8]) to evaluate their DIS efforts. We anticipate that access to the repository will encourage repeated use of the same, high-quality instruments to measure similar constructs across settings, reduce instrument redundancy, and increase the potential for the DIS field to evolve more rapidly. In addition to being a resource for existing DIS instruments, the repository may stimulate new areas of research and instrument development given that some constructs are saturated whereas others are lacking in instrumentation. Our preliminary results also signal a need for new instrumentation targeting non-provider stakeholders such as leaders and external change agents (e.g., implementation practitioners or intermediaries), particularly in light of research identifying the role they play in implementation success (e.g., [25]). The ongoing application of our evidence-based assessment rating criteria leads us to anticipate a dearth of high-quality, psychometrically sound instruments, which will signal a need for instrument development of greater quality.

Although the above suppositions represent more short-term implications, long-term implications of this review are twofold, at minimum. First, the application of the EBA rating criteria as described in step 6 will aid in identifying psychometrically strong instruments and a potential consensus battery of high-quality, essential DIS instruments as a basic resource for researchers and stakeholders to advance cross-study comparisons. Second, it is our intention that the SIRC repository will be a dynamic resource. That is, the repository will grow with the evidence base to incorporate newly developed and/or tested instruments, as well as instruments identified via methodologies of colleagues completing relevant research (e.g., crowd sourcing methods). We believe this dynamic process will improve the efficiency and rigor of implementation science evaluations as a whole.

### Limitations

There are several noteworthy limitations inherent in this methodology. To ensure rigor and quality of the resulting repository, each step is meticulous and necessarily time-consuming and must be replicated by a second party. As a result, the intensity of time, resources, and personnel required by this comprehensive and multi-faceted methodology may be a potential limitation. Specifically, (a) initial literature reviews to identify instruments for targeted constructs take approximately 1.5–3 h, (b) cross-checking reviews take an additional 45 min–1 h, (c) instrument-specific literature reviews take an average of 2.5–4 h; (d) cross-checking instrument-specific literature reviews adds 1–3 h, and (e) rating requires an average of 50 min to complete. Because of limited funding, these preliminary results have taken roughly 2 years to achieve. It is highly encouraging, however, that the careful creation of project protocols and international support forthcoming for this project have allowed us to engage multiple core worksites and a large task force committed to realizing the goals of the SIRC IRP. Moreover, the lead authors (CCL, CS, and BJW) anticipate receiving grant funding from the National Institute of Mental Health to extend this work to also include pragmatic ratings of instruments, a critical domain for advancing the practice of implementation in real-world settings [26]. Another potential limitation of our work centers on the specific frameworks used to guide construct selection. Basing our work on the CFIR [7] and Implementation Outcomes Framework [8] provides a comprehensive conceptual framework, yet it is clear that DIS investigators employ diverse frameworks delineating unique constructs not included in the SIRC IRP [2]. Nonetheless, we are hopeful that the thoughtful selection of these comprehensive and complementary frameworks will identify and make accessible a range of high-quality instruments that will be relevant to the majority of interested researchers and stakeholders.

### Conclusions and future directions

This multi-faceted and collaborative methodology is perhaps the most comprehensive attempt to identify, evaluate, and synthesize DIS instruments to date. Moving forward, we will review literature as it is published to ensure that this repository evolves with developing research, hence the need for a website platform. We have assigned a research assistant to review the Implementation Network monthly e-newsletter for additional instruments of relevance to our comprehensive review. In addition, a function for setting Google Scholar alerts according to our search strings will be implemented to review research published on a weekly basis to add relevant instruments and literature to our database.

In collaboration with our web master, we will design functionality to enable researchers and stakeholders to access, share (upload), and interact with the content. Researchers and stakeholders who are SIRC members^c^ will be able to access, contribute, and track the dynamic expansion of the repository, receive notifications when new instruments are rated and added, and will be encouraged to engage with the development efforts. In addition, the repository will have inherent functionality to invite researchers and stakeholders who access instruments to share their data with the “community”. Our long-term goal is to build a large, open-access dataset ripe for the application of more complex analyses of the instruments’ psychometric properties. The results of the rigorous SIRC Instrument Review Project methodology will position the field to engage in careful evaluation of DIS efforts. The resulting decision aid with head-to-head graphical comparisons of instrument qualities will facilitate instrument identification and selection with open access and position researchers and stakeholders to employ psychometrically validated instruments and contribute to focused instrument development efforts.

### Endnotes

^a^Implementation science refers to the scientific study of strategies used to integrate evidence into real-world settings [27]. Implementation practice is the act of integrating evidence into real-world settings [28]. Instrument, in the case of this project, refers to quantitative tools, surveys, or measures that can be administered to individuals to obtain perspectives or information regarding their experience. Psychometric properties refer to outcomes of psychological testing of an instrument that reflects how well it measures a construct of interest with respect to reliability and validity.

^b^Instrument Review Task Force members listed in alphabetical order: Drs. Gregory Aarons, Cassidy Arnold, Melanie Barwick, Rinad Beidas, Helen Best, Elisa Borah, Craig Bryan, Adam Carmel, Mark Chaffin, Kate Comtois, Laura Damschroder, Dennis Donovan, Shannon Dorsey, Michelle Duda, Julia Felton, Dean Fixsen, Howard Goldman, Carmen Hall, Rochelle Hanson, Petra Helmond, Amanda Jensen-Doss, Sarah Kaye, Meghan Keough, Sara Landes, Cara Lewis, Marsha Linehan, Aaron Lyon, Michael McDonell, Kate McHugh, Maria Mancebo, Shari Manning, Christopher Martell, Erin Miga, Brian Mittman, Sandra Naoom, Byron Powell, Raphael Rose, Lisa Ruble, Joe Ruzek, Anju Sahay, Sonja Schoenwald, Rebecca Selove, Jeffrey Smith, Cameo Stanick, Bradley Steinfeld, Phil Ullrich, Elizabeth A. Wells, and Shannon Wiltsey Stirman.

^c^Anyone can register to be a SIRC member at seattleimplementation.org and thus have access to the repository.

## Additional file

Additional file 1:
**Search string parameters for literature review.** Standard search string parameters developed for the construct literature reviews.

## Abbreviations

CFIR: Consolidated Framework for Implementation Research
DIS: Dissemination and Implementation Science
DoI: Diffusion of innovations
EBA: Evidence-based assessment
GEM: Grid-Enabled Measures Project
NIH: National Institutes of Health
PARiHS: Promoting Action in Health Services Framework
PRISM: Practical Robust Implementation and Sustainability Model
RA: Research assistant
SIRC: Society for Implementation Research Collaboration (formerly known as Seattle Implementation Research Conferences)
SIRC IRP: SIRC Instrument Review Project
