# Correction to: The Society for Implementation Research Collaboration Instrument Review Project: a methodology to promote rigorous evaluation

**DOI:** 10.1186/s13012-019-0963-6

**Published:** 2020-01-03

**Authors:** Cara C. Lewis, Cameo F. Stanick, Ruben G. Martinez, Bryan J. Weiner, Mimi Kim, Melanie Barwick, Katherine A. Comtois

**Affiliations:** 1 Department of Psychological and Brain Sciences, Indiana University, 1101 E. 10th St, Bloomington, IN 47405 USA; 2 Department of Psychiatry and Behavioral Sciences, Harborview Medical Center, School of Medicine, University of Washington, Box 359911, 325 9th Ave, Seattle, WA 98104 USA; 3 Department of Psychology, University of Montana, 32 Campus Dr., Skaggs Bldg. 362, Missoula, MT 59812 USA; 4 University of North Carolina at Chapel Hill, 1102-C McGavran-Greenberg Hall, 135 Dauer Drive, Campus Box 7411, Chapel Hill, NC 27599-7411 USA; 5 North Carolina Translational and Clinical Sciences Institute, University of North Carolina at Chapel Hill, 160 N. Medical Drive, Brinkhous-Bullitt, 2nd Floor, CB# 7064, Chapel Hill, NC 27599-7064 USA; 6 Child Health Evaluative Sciences, Research Institute, The Hospital for Sick Children, 555 University Avenue, Toronto, ON M5G 1X8 Canada; 7 Department of Psychology, Virginia Commonwealth University, 806 West Franklin St, Richmond, VA 23220 USA

**Correction to: Implementation Science (2015) 10:2.**

**https://doi.org/10.1186/s13012-014-0193-x**

Following publication of the original article [1] the authors reported an important acknowledgement was mistakenly omitted from the ‘Acknowledgements’ section. The full acknowledgement is included in this Correction article:

**Acknowledgements**

The preparation of this manuscript was supported, in kind, through the National Institutes of Health R13 award entitled, “Development and Dissemination of Rigorous Methods for Training and Implementation of Evidence-Based Behavioral Health Treatments” granted to PI: KA Comtois from 2010 to 2015. Dr. Bryan J. Weiner’s time on the project was supported by the following funding: NIH CTSA at UNC UL1TR00083. We would also like to acknowledge the numerous undergraduate research assistants (RAs) who contributed countless hours to this project. Indiana University RAs listed in alphabetical order: Hayley Ciosek, Caitlin Dorsey, Dorina Feher, Sarah Fischer, Amanda Gray, Charlotte Hancock, Hilary Harris, Elise Hoover, Taylor Marshall, Elizabeth Parker, Paige Schultz, Monica Schuring, Theresa Thymoski, Lucia.

Walsh, Kaylee Will, Rebecca Zauel, Wanni Zhou, Anna Zimmerman, and Nelson Zounlome. University of Montana RAs (undergraduate and graduate) listed in alphabetical order: Kaitlyn Ahlers, Sarah Bigley, Melina Chapman, May Conley, Lindsay Crosby, Bridget Gibbons, Eleana Joyner, Samantha Moore, Julie Oldfield, Kinsey Owen, Amy Peterson, and Mark Turnipseed.

University of North Carolina RAs: Emily Haines and Connor Kaine.

In addition, the authors would like to acknowledge Lindsay Crosby (now Dr. Lindsay Meyer) who contributed significant writing portions to this manuscript.
