# Supplementary material for: The Society for Implementation Research Collaboration Instrument Review Project: A methodology to promote rigorous evaluation
Source: Implement Sci. 2015 Jan 8;10:2. doi: 10.1186/s13012-014-0193-x (PMC4308900; doi:10.1186/s13012-014-0193-x)
Supplement: Additional file 1: — Search string parameters for literature review. Standard search string parameters developed for the construct literature reviews. [file 13012_2014_193_MOESM1_ESM.docx]

**Table S1** Search string parameters for literature review

| 1. (implement* OR adopt* OR “quality improv*” OR diffus* OR disseminat*) |
| --- |
| AND |
| 2. (“empirically supported treatment” OR “evidence based practice” OR “evidence based treatment” OR “evidence based intervention” OR innovation OR guideline) |
| AND |
| 3. ( measure OR instrument OR survey OR questionnaire) |
| AND |
| 4. (“mental health” OR “behavioral health” OR “behavioural health” OR health) |
| AND |
| 5. (construct OR synonym) |
| e.g., (feasibility OR transferability OR applicability OR practicability OR workability) |

Asterisk tells the search engine to return alternate spellings for a word at the point that the asterisk appears.
